# Timmy’s Trip to Planet Earth: The Long-Term Effects of a Social and Emotional Education Program for Preschool Children

**DOI:** 10.3390/children12080985

**Published:** 2025-07-26

**Authors:** Valeria Cavioni, Elisabetta Conte, Carmel Cefai, Veronica Ornaghi

**Affiliations:** 1 Department of Humanities and Social Sciences, Faculty of Society and Communication Sciences, Universitas Mercatorum, 00186 Rome, Italy; valeria.cavioni@unimercatorum.it; 2 Department of Human and Social Sciences, University of Bergamo, 24129 Bergamo, Italy; 3 Department of Psychology, University of Malta, MSD 2080 Msida, Malta; carmel.cefai@um.edu.mt; 4 Department of Human Sciences for Education “Riccardo Massa”, University of Milano-Bicocca, 20126 Milan, Italy; veronica.ornaghi1@unimib.it

**Keywords:** social and emotional education, preschool intervention, school transition, emotion comprehension, prosocial behavior, longitudinal study, social competence

## Abstract

Background/Objectives. Social and Emotional Education (SEE) interventions during early childhood have shown considerable promise in enhancing children’s emotion understanding, social competence, and behavioural adjustments. However, few studies have examined their long-term impact, especially across the preschool-to-primary school transition. This study evaluated the effectiveness of a manualized SEE program, Timmy’s Trip to Planet Earth, in promoting emotional, behavioural, and social functioning over time. Methods. A quasi-experimental longitudinal design was adopted with pre- and post-test assessments conducted approximately 18 months apart. Participants were 89 typically developing children (aged 59–71 months), assigned to an experimental group (*n* = 45) or a waiting-list group (*n* = 44). The program combined teacher training, classroom-based lessons, home activities, and teachers’ ongoing implementation support. The effectiveness of the program was measured via the Test of Emotion Comprehension (TEC), the Strengths and Difficulties Questionnaire (SDQ), and the Social Competence and Behavior Evaluation (SCBE-30). Results. Significant Time × Group interactions were observed for the TEC External and Mental components, indicating greater improvements in emotion recognition and mental state understanding in the intervention group. The SDQ revealed significant reductions in conduct problems and increased prosocial behaviours. In the SCBE-30, a significant interaction effect was found for social competence, with the intervention group showing greater improvement over time compared to the control group. Conclusions. The findings suggest that SEE programs can produce meaningful and lasting improvements in children’s emotional and social skills across key educational transitions. Teacher training and family involvement likely played a critical role in supporting the program’s sustained impact.

## 1. Introduction

Children’s early social and emotional competencies are critical predictors of later academic achievement, mental health, and positive classroom engagement [1,2]. These skills are essential for children to build and sustain peer relationships, adapt to classroom norms, and engage effectively in learning activities [3,4]. The preschool years represent a critical developmental period during which children begin to acquire foundational skills that support both social functioning and early academic development. These include mantaining attention, persisting with tasks, and collaborating effectively with teachers and peers in structured educational activities [5,6].

Research has shown that preschoolers with higher levels of emotional competence tend to exhibit fewer externalizing behaviours, experience greater social inclusion, and achieve stronger learning outcomes [7,8]. In contrast, children with difficulties in emotion regulation are more likely to experience peer conflict, reduced attention during class activities, and challenges with teacher relationships [9,10].

Numerous studies have demonstrated that preschool-based social and emotional education programs are effective in enhancing young children’s emotional understanding, social competence, and behavioural adjustment [11,12]. These early interventions aim to build foundational skills that support positive developmental outcomes across social, emotional, and academic domains [13,14].

However, despite this well-established short-term effectiveness, relatively few longitudinal studies have examined the extent to which these early benefits are sustained over time [15], particularly during key educational transitions such as the move from preschool to primary school [16,17]. This remains a crucial gap in the literature, considering that early promotion of social and emotional competencies plays a vital role in fostering school readiness and supporting children’s long-term academic and social adjustment [18].

## 2. Examining the Critical Transition from Preschool to Primary Education

The transition from preschool, typically attended by children between the ages of three and five, to formal primary education around age six, represents a key developmental phase characterized by profound changes in learning environments, pedagogical expectations, and social dynamics. Children move from a flexible, play-based context that emphasizes autonomy, emotional expression, and peer collaboration, to a more structured, teacher-directed setting where academic outcomes, seat time, and content-specific instruction are prioritized [19]. This shift often poses substantial challenges, requiring children to adapt to new routines, roles, and behavioural expectations. Even those who appear well-prepared may exhibit increased internalizing or externalizing behavior problems during this adjustment period [20,21].

Empirical research underscores the significance of this early educational transition, both in the short and long term. Immediately following school entry, children struggling to adjust frequently show elevated anxiety levels, reduced classroom engagement, and emerging academic difficulties [22,23]. When unaddressed, these initial challenges may lead to long-lasting effects, including poorer socio-emotional functioning, increased absenteeism, and widening academic disparities over the course of primary education [24]. Consequently, this period is widely recognized as a sensitive developmental window in which timely interventions can leverage the brain’s plasticity to strengthen children’s self-regulation, social skills, and early academic readiness [25].

There is broad international agreement that the transition to primary school is a complex, multidimensional process involving shifts in physical, relational, and cultural contexts. Its destabilizing potential is amplified by the fact that it coincides with the rapid development of children’s executive functions, emotion regulation, and emerging social identities [26]. Notably, research indicates that nearly half of all children experience moderate to serious adjustment difficulties during this transition [27].

The rise in behavioural difficulties at school entry can be understood as a response to heightened demands on socio-emotional skills at a time when these competencies are still consolidating [28]. Early primary school requires greater self-regulation, adherence to classroom norms, and effective peer interaction, skills that many children have not yet fully developed [29]. In many cases, behavioural concerns commonly attributed to temperament or family background may instead reflect a misalignment between the developmental capacities of the child and the expectations of the new educational setting [30,31].

This evidence points to the urgent need for proactive, developmentally informed interventions. Targeted social and emotional education programs implemented during the final preschool year have been shown to reduce transition-related stressors, foster adaptive coping, and promote self-regulation and prosocial behaviour [32,33]. Such interventions are particularly effective when they are embedded within the curriculum and supported by teacher professional development in fostering children’s relational and emotional competencies [34].

Furthermore, increasing evidence suggests that the timing of interventions is critical [35]. The final year of preschool represents an optimal window for scaffolding emotional competence and social skills that predict positive school trajectories [36]. However, benefits are maximized when these interventions are coupled with sustained monitoring into the first year of primary school. Such monitoring allows educators to identify persistent difficulties and adjust support accordingly [37,38].

Ultimately, the transition to primary school should be understood as a systemic and relational process shaped by the interconnected roles of schools, families, and communities [39]. Smooth transitions depend on relational continuity, shared expectations, and coordinated planning between preschool and primary educators, alongside meaningful family engagement [40]. Consequently, intervention programs should strongly consider taking into account the child’s life beyond the school setting, acknowledging that young children’s developmental outcomes are greatly influenced by the quality of support in their home environments.

## 3. The Social and Emotional Education Framework

The Social and Emotional Education (SEE) framework [41] provides a comprehensive framework for promoting children’s social and emotional development within educational contexts. The framework is grounded in six complementary theoretical perspectives: social and emotional learning, positive psychology, resilience theory, mindfulness education, inclusive education, and caring classroom communities.

The first foundational pillar is social and emotional learning (SEL), defined as the process through which individuals acquire and apply the knowledge, skills, and attitudes to develop healthy identities, manage emotions and achieve personal and collective goals, feel and show empathy for others, establish and maintain supportive relationships, and make responsible and caring decisions [42]. SEL offers the conceptual and structural backbone of the SEE model, outlining a developmental roadmap of core social, emotional, and cognitive competencies that underpin children’s well-being, learning, and positive school adjustment [43].

The second key influence is positive psychology, which emphasizes strengths-based and preventive approaches to promote human flourishing [44]. Rather than focusing on deficits or dysfunctions, this perspective prioritizes the development of character strengths such as gratitude, perseverance, and empathy—qualities shown to enhance motivation, academic achievement, and resilience [45]. Positive psychology school-based interventions have proven effective in increasing life satisfaction and reducing distress, making them well-suited for school-based implementation [46].

Resilience theory adds a systemic and developmental dimension to the framework. It conceptualizes resilience not as a fixed trait, but as a dynamic process emerging from interactions among individual, relational, and contextual protective factors [47,48]. Resilience is nurtured both by strengthening children’s coping resources and by fostering environments, such as classrooms and schools, that promote connection, inclusion, and emotional safety [49].

The fourth theoretical lens is mindfulness education, which supports the cultivation of attention regulation, emotional awareness, and self-reflection [50]. Mindfulness practices have been shown to reduce stress and internalizing symptoms while enhancing self-regulation, attention, executive function, and social and prosocial behaviours in children [51].

Inclusive education represents a crucial ethical and pedagogical dimension to SEE. It advocates for the right of all children, regardless of background, ability, or need, to fully participate in equitable and emotionally supportive learning environments [52]. Within SEE, inclusive education ensures that emotional and social learning opportunities are accessible to all students and embedded in a climate of respect, empathy, and cultural sensitivity [53,54].

Finally, the concept of caring classroom communities emphasizes the relational foundation of learning. This perspective highlights the central role of warm, respectful relationships in promoting children’s sense of belonging, psychological well-being, and moral development [55]. A caring classroom climate fosters prosocial behaviour, emotional engagement, and collaborative learning, while reducing conflict, school avoidance, and social exclusion [56].

**Figure 1:**
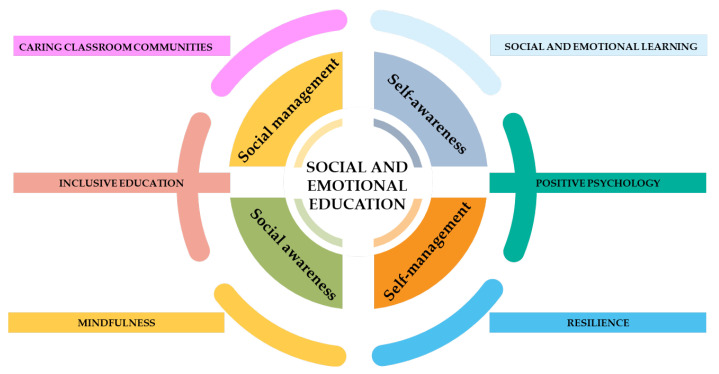
Theoretical framework of Social and Emotional Education. This figure illustrates the conceptual framework of Social and Emotional Education [41]. At the core of the model are four areas of competence: self-awareness, self-management, social awareness, and social management. These competencies are underpinned by six complementary theoretical approaches: social and emotional learning, positive psychology, resilience theory, mindfulness education, inclusive education, and caring classroom communities.

Anchored in these six perspectives, the SEE model organizes children’s competencies into four interconnected developmental areas (Figure 1).

The first area is self-awareness, which refers to the ability to accurately recognize one’s own emotions, thoughts, and values, and to understand how these internal states influence behaviour. This includes recognizing emotional states, developing self-concept, and becoming aware of personal strengths and limitations.

The second area is self-management, defined as the ability to regulate one’s emotions, thoughts, and behaviours across different situations. It includes impulse control, stress management, perseverance, and the capacity to set and pursue personal goals. Effective self-management is seen as foundational for both learning and social integration.

The third area, social awareness, involves the ability to take the perspective of others and to empathize with individuals from diverse backgrounds and cultures. It encompasses an understanding of social norms, sensitivity to others’ feelings, and the development of moral reasoning in interpersonal contexts.

Finally, the fourth area is social management, which encompasses the skills needed to establish and maintain healthy, constructive relationships. These include communication, cooperation, negotiation, conflict resolution, and responsible decision-making in social settings. Through this domain, children learn how to actively participate in group life and contribute to prosocial and inclusive communities.

The SEE framework has received substantial institutional recognition at the European policy level. Initially highlighted in the landmark NESET report *Strengthening social and emotional education as a core curricular area across the EU* [57], the SEE framework has been identified as a key reference framework for promoting well-being, equity, and inclusion in schools. This recognition was reaffirmed in a later NESET report by the European Commission [58], as well as in the more recent European guidelines for supporting well-being at school [59], both of which underscore the need to integrate social and emotional education within national education frameworks. The framework was cited as a reference for informing teacher professional development, curriculum innovation, and whole-school mental health promotion, especially in the context of increasing psychosocial risks and post-pandemic challenges [57,58].

### Aims of the Study

Building on the theoretical foundations outlined above, the present study aimed to evaluate the long-term effectiveness of the SEE-based program *Timmy’s Trip to Planet Earth: A Self and Social Adventure*, implemented during the final year of preschool. The primary objective was to assess whether the positive effects of the intervention were sustained 18 months after its completion, at the end of the children’s first year of primary school. More specifically, this study seeks to address a critical gap in existing literature by evaluating the long-term impact of a SEE program delivered in early childhood.

Although previous research has demonstrated the short-term benefits of SEE interventions, there remains a notable paucity of studies examining whether these effects are maintained over time, particularly during the sensitive developmental period marked by the transition from preschool to primary education. This transitional phase is characterized by increased cognitive, emotional, and social demands, making it essential to assess the durability of socio-emotional gains beyond the immediate post-intervention period. Furthermore, the present study is distinguished by its adoption of a rigorous multi-informant evaluation strategy, which combines standardized direct assessments of children’s emotional understanding with teacher-reported observations of prosocial and social behavior and socio-emotional difficulties. In addition, the intervention was delivered directly by classroom teachers who participated in targeted professional training sessions and received ongoing supervision throughout the program’s implementation.

The study tested the following hypotheses:

**H1:**  *Children who participated in the intervention were expected to show significantly greater improvements in emotional understanding from pre-test to post-test compared to children in the control group*;

**H2:**  *Children in the experimental group would exhibit significantly lower levels of emotional and social difficulties and significantly higher levels of prosocial behaviour*;

**H3:**  *Children in the experimental group would display significantly better outcomes in terms of social competence, lower levels of internalizing and externalizing behaviours relative to their peers in the control group.*

## 4. Materials and Methods

### 4.1. Participants

A total of 96 five-year-old children attending the final year of preschool were approached for participation in the study. Schools were recruited using a convenience sampling approach. An open invitation to participate in the study was disseminated via institutional emails and social media platforms. Only schools that voluntarily responded and agreed to participate were included in the study. Informed consent was obtained from all parents or legal guardians, and all 96 children for whom consent was requested took part in the pre-test phase, conducted at the beginning of the school year. Children were distributed across nine classes in two public preschools located in Northern Italy. To ensure impartial allocation, the research team randomly assigned each school to either the experimental group or the waiting-list control group through a manual draw. As a result, all classes in one preschool implemented the intervention, while those in the other preschool served as the control waiting list group and received the program only after the post-test phase.

The participating classes (five in the experimental school and four in the control school) reflected the typical Italian mixed-age preschool model, including children aged three to five. In line with the school’s educational plan and national principles of educational inclusion, all children in these classes participated in the classroom-based activities. However, for research purposes, only the five-year-old children were assessed at pre- and post-test, as they were in their final year of preschool and approaching the transition to primary school.

The post-test was conducted approximately 18 months later, at the end of the children’s first year of primary school. The final sample considered in the present study consisted of 89 children (46 boys and 43 girls) who completed both the pre-test and post-test assessments. Only children with data from both time points were included in the analyses. Sample attrition occurred due to typical factors such as transfers to other schools, absence during the data collection period, or the withdrawal of parental consent. All participants were typically developing children.

The study was approved by the Ethics Committee of the University of Milano-Bicocca and conducted following the ethical standards outlined in the Declaration of Helsinki.

### 4.2. Design and Intervention Description

The study employed a quasi-experimental longitudinal design, comparing an experimental group with a control group through assessments conducted approximately 18 months apart. The research was organized into three phases: pre-test, intervention, and post-test. The pre-test was administered at the beginning of the children’s final preschool year, before the intervention, while the post-test was conducted at the end of their first year in primary school. This extended timeline enabled the examination of long-term effects across a critical developmental transition.

Children in the experimental group took part in *Timmy’s Trip to Planet Earth: A Self and Social Adventure*, a structured program designed to translate the principles of the SEE framework into practice [41]. Meanwhile, the control group followed the standard curriculum without exposure to any SEE-based content during the study period.

Table 1 provides an overview of the program’s learning objectives, organized according to the four core areas of the SEE framework.

The intervention consisted of four core components: (a) initial teacher training, (b) delivery of a classroom-based curriculum, (c) implementation of complementary home-based activities, and (d) ongoing supervision and support for educators.

The first component of the intervention was a preliminary teacher training session, lasting approximately eight hours, delivered by researchers with expertise in SEE. The training involved all educators working in the classrooms selected for the study, including both main classroom teachers and, where present, additional support teachers assigned to children with special educational needs. The lead classroom teacher in each class was primarily responsible for implementing the activities with the children during the intervention phase. The training was designed to strengthen teachers’ emotional and social competence to prepare them to create emotionally supportive and inclusive learning environments. The training combined theoretical foundations with hands-on practice, ensuring that educators could guide children through the proposed activities in a developmentally appropriate and emotionally responsive manner. Particular emphasis was placed on promoting emotional safety, active participation, and prosocial peer interactions. In addition, the sessions addressed strategies for managing challenging behaviours and fostering constructive teacher–student relationships, recognizing that teachers’ social-emotional skills play a critical role in classroom climate, students’ behavioural adjustment, and academic engagement [60]. This inclusive and whole-school approach aimed not only to equip educators with the theoretical knowledge and practical tools necessary to deliver the program, but also to promote their own social and emotional competencies.

Supporting teachers in developing their own social and emotional competence is essential for the sustainability of manualized SEE programs. Research indicates that when educators feel emotionally equipped and confident, they are more likely to implement SEE interventions with fidelity and responsiveness, adapt practices to student needs, and benefit themselves from improved job satisfaction and well-being [61,62,63].

The second component consisted of a classroom-based curriculum composed of 24 structured sessions, implemented weekly by teachers. The program was seamlessly integrated into the regular school schedule and delivered over approximately six months, from December to June, following the pre-test phase and aligning with the remainder of the academic year.

The sessions were structured around the story of Timmy, a small alien robot who, after an accidental journey through space, lands on Earth and begins engaging with children and adults in everyday social and emotional situations. The narrative framework was intentionally selected to foster children’s engagement, support identification with the characters, stimulate perspective-taking, and promote emotional reflection through symbolic play and storytelling.

Each session began with a brief mindfulness-based activity, lasting approximately 5 to 7 min, designed to help children focus their attention, regulate arousal, and become more aware of their internal states, thereby creating a supportive emotional climate for learning. The sessions, each lasting about one hour, were thematically anchored in the unfolding narrative of *Timmy’s Trip to Planet Earth*. Some included a narrated story segment, followed by comprehension and expressive tasks such as drawing, discussion, or dramatization. Others, while not featuring a new reading, proposed practical activities, such as cooperative games, motor tasks, or role-play, that extended and deepened the emotional and social themes introduced earlier in the storyline. By using Timmy’s unfamiliarity with Earthly emotions and relationships as a narrative device, the program supported children in externalizing and making sense of their own emotional experiences. All sessions were designed to reinforce the social and emotional content embedded in the program.

**Figure 2:**
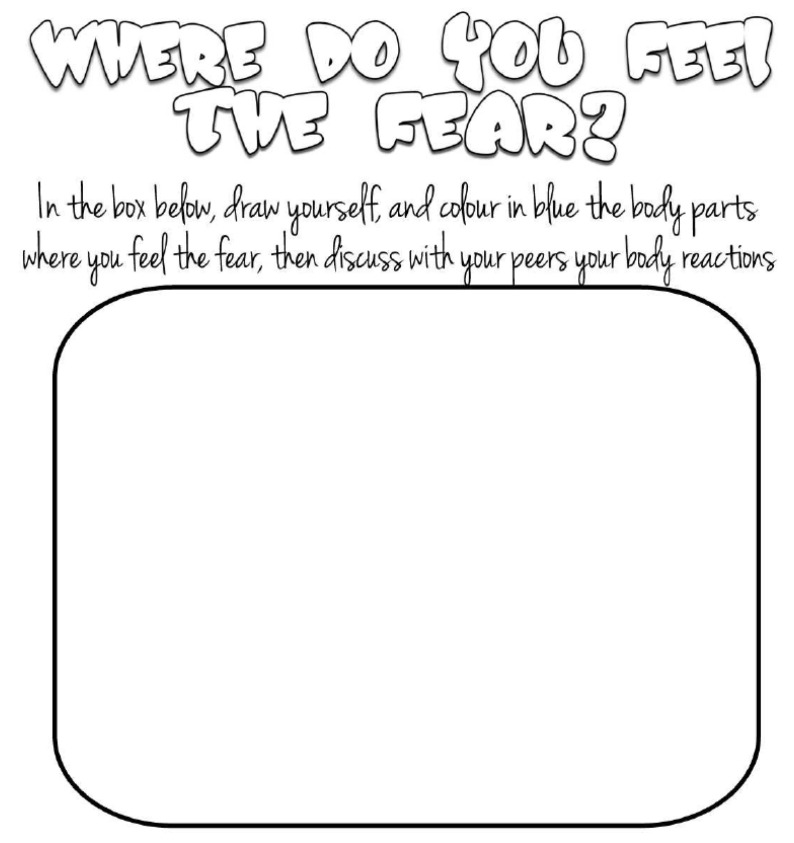
The image provides an example of an activity from the second core area of the SEE framework, specifically focused on self-management. The activity invites children to reflect on how fear is experienced in their bodies. In the provided worksheet, children are instructed to draw themselves inside the outlined box and to colour in blue the body parts where they feel fear. They are then encouraged to discuss their bodily reactions with peers. The primary objective of this activity is to promote emotional and bodily awareness, supporting children in recognising the physical sensations associated with fear and providing strategies for self-regulation.

The activities were evenly distributed across the four core areas of the SEE framework: self-awareness, self-management, social awareness, and social management (e.g., Figure 2). Each activity was outlined in a standardized manual provided to teachers, allowing for consistent implementation across classrooms while maintaining fidelity to the program model.

The third component consisted of complementary home-based activities aimed at promoting the generalization of skills beyond the classroom and fostering collaboration between schools and families. Following each classroom session, children received a take-home worksheet summarizing the goals of the activity and proposing a brief task to be completed jointly with their caregivers. These home activities mirrored the classroom content and targeted the same social-emotional competencies. Each worksheet followed a structured format, beginning with a brief explanation of the school-based activity, followed by an engaging parent–child interaction such as a drawing, cooperative game, cartoon, or simple worksheet. These tasks were designed to encourage emotional dialogue within the family and typically required about 30 minutes to complete [64].

The fourth and final component involved ongoing supervision and support for participating educators throughout the program’s implementation. Three supervision sessions, each lasting approximately two hours, were organized during the school year. These meetings served multiple purposes: they ensured fidelity of implementation, addressed any emerging questions or challenges raised by teachers, and provided a reflective space for discussing both pedagogical and emotional aspects of the intervention. This ongoing support played a key role in maintaining teacher engagement, program quality, and alignment with the SEE framework.

The control group continued with the standard educational curriculum and routine classroom activities provided by the preschool, without any exposure to the intervention. Teachers in the control group did not receive any additional training or support during the study period. This allowed for a clear comparison between the effects of the intervention and typical educational practice. After the post-test, they mirrored the full experience of the intervention group. First, teachers received the initial training as the experimental group, enriched by the feedback and reflections gathered during the earlier implementation with the experimental group. The training sessions gave educators the knowledge and practical tools to implement the activities in their classes. In the next phase, they delivered the classroom-based curriculum, while parents implemented complementary home-based activities at home with children. In parallel, teachers received ongoing supervision and support.

### 4.3. Measures

A multi-informant approach was employed, integrating direct assessments of children’s emotional understanding with teacher-reported evaluations of their socio-emotional difficulties, prosocial behaviours, and social competence.

#### 4.3.1. Demographic Information

Demographic data were collected from parents using an anonymous questionnaire. Each child was assigned a unique alphanumeric code to ensure anonymity while allowing linkage across different data sources, including teacher-reported measures and direct child assessments. The demographic form included information such as the child’s gender and age.

#### 4.3.2. Emotion Understanding

This domain was assessed by the Test of Emotion Comprehension [65] in its Italian version [66], administered by trained researchers who were blinded to the experimental condition of the children being assessed. The TEC is a standardized instrument designed to assess emotional understanding in children aged 3 to 11 years. It evaluates nine interrelated components of emotion comprehension: recognition of facial expressions, understanding of external situational causes, desires, beliefs, memories, regulation strategies, control of emotional expression, mixed emotions, and moral emotions. These components reflect a developmental progression in children’s ability to interpret and reason about emotional experiences.

The TEC consists of an A4-format picture book. Each page presents a schematic vignette illustrating a social scenario with a character whose face is left blank. Children are asked to choose the appropriate emotional expression, depicted in four facial options at the bottom of the page, that best fits the narrative presented by the researcher. The test focuses on basic emotions: happiness, sadness, anger, fear, and a neutral state. The assessment is administered individually and takes approximately 15 to 20 min. In its Italian validation, the TEC showed adequate internal consistency [65]. Further support for the instrument’s psychometric properties has been provided by recent studies (e.g., [67,68]). In the present study, emotion comprehension was analyzed based on these three composite dimensions: (1) the External dimension, comprising emotion recognition, understanding of external causes, and memory; (2) the Mental dimension, including the roles of desires, beliefs, and expression control; (3) the Reflective dimension, involving emotion regulation, mixed emotions, and moral emotions. In this study, Cronbach’s alpha coefficients for the three TEC dimensions ranged from 0.72 to 0.78, indicating acceptable internal consistency.

#### 4.3.3. Socio-Emotional Difficulties and Prosocial Behaviours

Children’s socio-emotional difficulties and prosocial behaviours were assessed by the classroom teacher using the Italian version of the Strengths and Difficulties Questionnaire (SDQ) [69,70]. The SDQ is a 25-item instrument designed to assess children’s adjustment, targeting individuals aged 4 to 16 years. Items are rated on a three-point Likert scale (0 = not true, 1 = somewhat true, 2 = certainly true) and are organized into five subscales: Emotional Problems (e.g., “Often complains of headaches, stomach-aches or sickness”), Conduct Problems (e.g., “Often has temper tantrums or hot tempers”); Hyperactivity (e.g., “Restless, overactive, cannot stay still for long”), Peer Problems (e.g., “Rather solitary, tends to play alone”), and Prosocial Behaviour (e.g., “Considerate of other people’s feelings”). Higher scores on the first four subscales indicate greater behavioural difficulties, while higher scores on the Prosocial Behaviour subscale reflect a greater frequency of altruistic and helping behaviours toward peers and adults. The SDQ demonstrated good internal consistency [71]. In the present study, internal consistency coefficients for the subscales ranged from 0.68 to 0.72.

#### 4.3.4. Social Competence

The Italian version [72] of the Social Competence and Behavior Evaluation Scale (SCBE; ref. [73]) was used to assess social behaviours. The SCBE is a standardized teacher-report instrument designed to assess emotional adjustment, peer interactions, and adult-child relational behaviours in children aged 30 to 78 months. The SCBE consists of 80 items rated on a 6-point Likert scale (1 = almost never to 6 = almost always), which capture the typical affective expression and emotion regulation strategies children display in the context of social interactions, particularly in classroom settings.

The SCBE is composed of three factors: Social Competence, Internalizing Problems, Externalizing Problems, and General Adjustment. Higher scores on the Social Competence scale reflect well-adjusted, flexible, and prosocial behaviours. For example, children with high scores in this domain are typically responsive, emotionally expressive in adaptive ways, and positively engaged with peers and adults (e.g., “Enjoys showing that he/she has learned new songs, games, and other things”). Conversely, elevated scores on the Internalizing Problems scale are indicative of anxious, withdrawn, or inhibited behaviours, such as excessive fatigue, low activity levels, and social disengagement (e.g., “Inactive, watches other children play”). High scores on the Externalizing Problems scale suggest the presence of disruptive behaviours, including oppositionality, impulsivity, and difficulty managing negative emotions in socially acceptable ways (e.g., “Cries when parents leave”). The Italian validation of the SCBE has demonstrated strong psychometric properties, with Cronbach’s alpha coefficients ranging from 0.84 to 0.94 [72]. In the current sample, internal consistency was also satisfactory, with Cronbach’s alpha values ranging from 0.74 to 0.87 across the three factors.

### 4.4. Data Analysis Strategies

All statistical analyses were conducted using IBM SPSS Statistics version 28. Children’s pre- and post-test scores were matched using identification codes. Before conducting the analyses on the effectiveness of the intervention, standard data-cleaning procedures were applied. Missing data accounted for less than 1% of the total dataset; therefore, a listwise deletion method was applied, as recommended for minimal levels of missingness [74]. Additionally, the distribution of each study variable was assessed, and all skewness and kurtosis values fell within the acceptable range of −1 to +1, indicating normality.

## 5. Results

### 5.1. Demographic Characteristics and Preliminary Analyses

Table 2 reports the demographic characteristics of the participants by group at the pre-test phase.

Preliminary analyses were performed to examine potential baseline differences between the experimental and control groups. Independent samples t-tests were conducted on all pre-test variables to assess group equivalence before the intervention [75].

To assess the effectiveness of the intervention, a series of repeated-measures multivariate analyses of variance (MANCOVAs) was conducted for each dependent variable. The analyses included Time (pre-test vs. post-test) as the within-subject factor, and Group (experimental vs. control) as the between-subject factor. The dependent variables consisted of the following: (a) the three dimensions of the TEC (External, Mental, and Reflective emotion understanding); (b) the five subscales of the SDQ (Emotional Problems, Conduct Problems, Hyperactivity, Peer Problems, and Prosocial Behaviour); and (c) the three dimensions of the SCBE-30 (Social Competence, Internalizing Behaviours, and Externalizing Behaviours). For all analyses, statistical significance was set at *p* < 0.05. Descriptive statistics (means and standard deviations) for all outcome variables at pre-test and post-test for both experimental and control groups are presented in Table 3.

Independent samples *t*-tests conducted on the pre-test scores revealed no statistically significant differences between the experimental and control groups across any of the measured variables, indicating that the two groups were equivalent in terms of emotional, behavioural, and social functioning prior to the implementation of the intervention.

### 5.2. Main and Interaction Effects

In this section, we present the main and interaction effects of the intervention on the variables assessed through the study measures. Specifically, results are reported for the following: (1) emotional understanding, as measured by the three TEC dimensions (External, Mental, Reflective); (2) social and emotional functioning, including emotional symptoms, conduct problems, hyperactivity, peer problems, and prosocial behaviours, assessed using the SDQ; and (3) dimensions of social competence, internalizing and externalizing behaviours, as measured by the SCBE-30.

Appendix A includes a summary of the results. Specifically, Table A1 reports the main effects for all outcome variables, while Table A2 presents the significant Group × Time interaction effects, including F-values, significance levels, and partial eta squared.

#### 5.2.1. Emotion Comprehension

Regarding the External component of the Test of Emotion Comprehension, the analysis revealed a significant main effect of Time, F(1, 87) = 7.77, *p* = 0.007, η^2^_p_ = 0.082, indicating that children’s ability to recognize emotions based on external cues improved significantly from pre- to post-test across both groups. Crucially, a significant Time × Group interaction was found, F(1, 87) = 5.44, *p* = 0.022, η^2^_p_ = 0.059, suggesting that the experimental group experienced significantly greater improvements than the control group.

In the Mental component, results showed a significant main effect of Time, F(1, 87) = 8.34, *p* = 0.005, η^2^_p_ = 0.087, reflecting an enhanced understanding of internal mental states (such as desires and beliefs) in both groups over time. The significant Time × Group interaction, F(1, 87) = 5.28, *p* = 0.024, η^2^_p_ = 0.057, further indicated that this improvement was substantially greater in children who received the intervention.

In the case of the Reflective component, the analysis yielded a significant main effect of Time, F(1, 87) = 3.94, *p* = 0.05, η^2^_p_ = 0.043, showing a general increase in children’s ability to reflect on complex emotional experiences across time. However, the Time × Group interaction was not significant, F(1, 87) = 0.75, *p* = 0.39, η^2^_p_ = 0.009, suggesting that both groups showed similar levels of improvement, with no additional benefit observed for the experimental group.

#### 5.2.2. Social and Emotional Functioning

The analysis of the Emotional Problems subscale showed no significant main effect of time, F(1, 87) = 3.28, *p* = 0.074, η^2^_p_ = 0.036, indicating that levels of emotional difficulties remained stable across time for both groups. The Time × Group interaction was also non-significant, F(1, 87) = 1.76, *p* = 0.189, η^2^_p_ = 0.020, suggesting that the intervention did not produce differential effects between the experimental and control groups in this domain.

In the Conduct Problems, the results revealed a significant main effect of time, F(1, 87) = 4.38, *p* = 0.039, η^2^_p_ = 0.048, indicating a general reduction in behavioural difficulties over time. Additionally, a significant Time × Group interaction was observed, F(1, 87) = 6.36, *p* = 0.014, η^2^_p_ = 0.068, with the experimental group showing a greater decline in conduct problems than the control group.

The analysis of Hyperactivity scores revealed no significant main effect of time, F(1, 87) = 2.87, *p* = 0.094, η^2^_p_ = 0.032, nor a significant Time × Group interaction, F(1, 87) = 0.02, *p* = 0.893, η^2^_p_ < 0.001. These results suggest that hyperactivity levels remained relatively unchanged over time and were not differentially affected by the intervention.

On the Peer Problems subscale, a robust main effect of time was found, F(1, 87) = 91.81, *p* < 0.001, η^2^_p_ = 0.513, indicating a substantial decrease in peer-related difficulties across both groups from pre-test to post-test. However, the interaction effect was not significant, F(1, 87) = 0.003, *p* = 0.959, η^2^_p_ < 0.001, implying similar improvements in both groups, regardless of intervention exposure.

Finally, the Prosocial Behaviour subscale showed a significant main effect of time, F(1, 87) = 6.23, *p* = 0.014, η^2^_p_ = 0.067, indicating an overall increase in prosocial behaviours over time. Crucially, a significant Time × Group interaction emerged, F(1, 87) = 18.26, *p* < 0.001, η^2^_p_ = 0.174, with the experimental group exhibiting a markedly greater improvement in prosocial behaviour compared to the control group.

#### 5.2.3. Social Competence, Internalizing and Externalizing Behaviours

The analysis of the Social Competence subscale revealed no significant main effect of Time, F(1, 87) = 0.001, *p* = 0.972, η^2^_p_ < 0.001, indicating that overall levels of social competence remained stable across the entire sample between pre-test and post-test. However, a significant Time × Group interaction emerged, F(1, 87) = 3.94, *p* = 0.050, η^2^_p_ = 0.043, suggesting that changes in social competence over time varied by group. Specifically, children in the experimental group showed a greater improvement in social competence following the intervention compared to those in the control group.

In the analysis of the Internalizing Problems, no significant main effect of Time was observed, F(1, 87) = 1.54, *p* = 0.218, η^2^_p_ = 0.017, indicating that internalizing behaviours such as anxiety or withdrawal remained relatively stable over time across both groups. Similarly, the Time × Group interaction was not significant, F(1, 87) = 1.95, *p* = 0.166, η^2^_p_ = 0.022, suggesting that the intervention did not differentially impact internalizing symptoms.

The analysis of Externalizing Problems revealed no significant main effect of Time, F(1, 85) = 0.49, *p* = 0.427, η^2^_p_ = 0.008, indicating no overall change in behaviours such as aggression or impulsivity from pre-test to post-test. The Time × Group interaction was likewise non-significant, F(1, 85) = 0.14, *p* = 0.709, η^2^_p_ = 0.002, showing no differential effects of the intervention on externalizing problems.

## 6. Discussion

The following discussion is structured around the main outcomes assessed by each measurement tool. Significant results are interpreted considering the study’s hypotheses regarding the effectiveness of the intervention.

### 6.1. Effects on Emotion Comprehension

The findings from the TEC analyses offer robust support for the long-term effectiveness of *Timmy’s Trip to Planet Earth* program in fostering emotional understanding in early childhood. Specifically, children in the experimental group demonstrated significantly greater improvements from pre-test to post-test across two of the three TEC components: External and Mental. These gains, observed 18 months after the end of the intervention, suggest sustained developmental benefits in emotion comprehension as a result of the SEE-based program.

The External component, which assesses children’s ability to recognize emotions from facial expressions, understand the emotional impact of external events, and link emotions to past experiences, showed a significant overall improvement over time in both groups. However, the significant Time × Group interaction indicated that the experimental group advanced more substantially, suggesting that the intervention specifically enhanced children’s capacity to accurately interpret and contextualize emotional cues in their environment.

Facial emotion recognition and the understanding of external causes of emotions are among the earliest emotional competencies to emerge in preschool years and are considered essential precursors to more complex emotional and social behaviours [18]. Several studies have demonstrated that preschoolers who show proficiency in interpreting facial expressions and situational emotional cues are better equipped to navigate peer interactions, exhibit greater empathy, and display lower levels of aggression and withdrawal [76,77,78].

In the current study, the effectiveness of the training in enhancing these external emotional competencies can be attributed to specific activities in *Timmy’s Trip to Planet Earth* program. For instance, activities within the self-awareness area included storytelling, picture recognition, and guided conversations in which children were asked to identify and label facial expressions in various social contexts. Additionally, home-based worksheets encouraged parent–child discussions about everyday emotional experiences, reinforcing classroom learning in the home environment [79,80].

Similarly, in the Mental component, which taps into more complex emotional understanding related to desires and beliefs, children in the intervention group exhibited a significantly greater increase over time compared to those in the control group. This suggests that the program was effective not only in fostering basic emotional awareness but also in enhancing children’s capacity to infer and reflect on mental states, which is commonly referred to as theory of mind [81]. Mental state reasoning plays a key role in steering social interactions, enabling children to predict others’ emotional responses based on underlying psychological causes such as intentions, goals, and false beliefs [82]. Research has consistently shown that theory of mind development is crucial for successful peer relationships and adaptive functioning in school contexts [83]. Specifically, the ability to coordinate multiple perspectives and understand divergent mental representations is strongly associated with prosocial behaviour, emotion regulation, and academic engagement during early childhood [84].

Within *Timmy’s Trip to Planet Earth* program, several activities were explicitly designed to foster children’s understanding of mental states, contributing to the observed improvements in the Mental component of the TEC. For example, during specific chapters of Timmy’s journey, such as those in which he encounters characters whose emotional reactions are shaped by their beliefs or desires, children were encouraged to reflect on how internal states influence emotional experiences. These narrative-based episodes were followed by role-play activities and guided discussions in which children were asked to infer and articulate the thoughts, beliefs, or intentions underlying each character’s emotions. In addition, educators facilitated metacognitive reflection by scaffolding children’s reasoning with targeted prompts and emotion labeling. This structured engagement with characters’ internal worlds likely enhanced children’s capacity for mental-state reasoning, a foundational skill for both emotional understanding and social interaction [85,86].

In contrast, the Reflective component, which assesses understanding of emotional regulation strategies, mixed emotions, and moral emotions, showed a significant main effect of time across the full sample, but the interaction effect was not significant. While all children appeared to improve in their reflective emotional understanding over time, the lack of a differential effect suggests that this more advanced level of emotional reasoning may be influenced by developmental maturation or general classroom experiences rather than the intervention alone. It may also indicate that longer training is needed to foster deeper moral and reflective competencies in young children, consistent with literature indicating that these abilities typically consolidate later in development [78].

Overall, these findings are consistent with previous research indicating that well-structured, evidence-based SEE interventions can significantly enhance children’s emotional understanding over time. For example, well-known SEE programs such as PROMEHS (Promoting Mental Health at Schools), implemented across various European contexts, have demonstrated the effectiveness of universal mental health interventions in promoting emotional competencies in early childhood, particularly when supported by whole-school implementation strategies and strong teacher involvement [79]. Similarly, our results align with those from the longitudinal evaluation of the Incredible Years program [80], which showed significant improvements in children’s self-regulation when interventions combined classroom-based curricula with teacher training in positive behavior management. Notably, both studies underscore the importance of embedding SEE within developmentally appropriate and emotionally rich activities, while also fostering collaboration between schools and families, principles that form the foundation of *Timmy’s Trip to Planet Earth*. This alignment suggests that long-term positive outcomes in children’s emotional competence are most likely when SEE programs are integrated into daily educational routines and actively supported by both educators and caregivers.

### 6.2. Effects on Social and Emotional Functioning

Among the five SDQ subscales, Conduct Problems and Prosocial Behaviour exhibited significant Time × Group interactions, suggesting specific improvements attributable to the intervention. In contrast, Emotional Symptoms, Hyperactivity, and Peer Problems showed no differential effects between groups, though in some cases, significant main effects of time were observed.

The significant reduction in Conduct Problems observed among children in the experimental group suggests that the intervention was effective in promoting behavioural self-regulation and reducing oppositional or disruptive behaviours. This outcome is consistent with the goals of *Timmy’s Trip to Planet Earth* program, which incorporates activities specifically designed to enhance self-regulation, particularly concerning emotions such as anger and fear which are two emotional states frequently associated with externalizing behaviours in early childhood. Difficulties in regulating anger can lead to impulsive reactions, defiance, and aggression, while unregulated fear may manifest as avoidance, hypervigilance, or oppositional behaviours as defensive strategies in perceived threatening contexts [87].

Throughout the program, Timmy encounters emotionally challenging situations that require him to recognize and manage intense feelings. For instance, in one narrative episode, Timmy experiences anger after being excluded by peers and learns strategies for expressing his feelings constructively rather than acting out. In another chapter, he must face a fear-inducing experience alone and discovers how to cope by seeking support and using calming techniques. These episodes serve as developmentally appropriate scenarios through which children can identify with the protagonist and explore their own emotional responses. Teachers guided children in reflecting on the emotional states of characters and the social consequences of different behavioural responses, thus creating opportunities to internalize prosocial norms and behavioural expectations. These findings are consistent with previous evidence showing that early interventions promoting emotional understanding and empathy are effective in reducing conduct-related difficulties in young children [80].

The Prosocial Behaviour scale revealed the most robust effect, showing a significant interaction in which children in the intervention group demonstrated a substantially greater increase in helping, sharing, and cooperative behaviours compared to the control group. This outcome is consistent with the explicit aims of *Timmy’s Trip to Planet Earth*, which emphasizes social awareness, empathy, and constructive interpersonal engagement as core developmental goals. Throughout the program, children engage with narrative episodes in which Timmy experiences everyday social situations such as attending a birthday party, overcoming fears, and offering or receiving help. These episodes emphasize the importance of mutual support and caring relationships. In the final part of the story, for example, Timmy is helped by a friend and the entire community to repair his spaceship so he can return home. This narrative reinforces key prosocial values, including cooperation, gratitude, and the importance of belonging to a supportive group. This narrative reinforces the importance of solidarity, reciprocity, and gratitude. These story-based experiences were further enhanced through collaborative classroom tasks and home-based reflections, creating opportunities for children to practice and internalize prosocial values. Such findings are in line with previous research demonstrating that well-designed SEE programs in early childhood can promote long-lasting improvements in prosocial behaviour by providing emotionally meaningful and contextually embedded learning opportunities [88].

In contrast, no significant Time × Group interactions were observed for Emotional Symptoms, Hyperactivity, and Peer Problems. The absence of the interaction effect on emotional symptoms is consistent with prior literature indicating that universal SEE programs, designed for all children regardless of risk level, often have limited impact on internalizing difficulties such as anxiety or sadness, which may require more targeted or intensive approaches to produce meaningful change [89]. Similarly, the lack of significant effects on hyperactivity is plausible given that participating children did not present with clinically elevated levels of attention or impulse-control difficulties, which are typically more resistant to change in the general population and often require individualized strategies for effective intervention [90].

Interestingly, peer-related difficulties significantly decreased over time in both groups, indicating a general improvement in peer relationships regardless of intervention exposure. This trend may reflect typical developmental progressions or the positive effects of school-based socialization processes, rather than being uniquely driven by the program. These findings are consistent with previous research showing that the ability to manage relationships with peers tends to increase with age and through continued participation in structured educational environments that promote cooperative interaction and social learning [11,91].

### 6.3. Effects on Social Competence, Internalizing and Externalizing Behaviours

The significant Time × Group interaction observed for social competence indicates that children who participated in the intervention demonstrated a more substantial improvement in their ability to interact effectively with peers and adults, compared to those in the control group.

The *Timmy’s Trip to Planet Earth* program incorporated a range of structured activities specifically designed to foster the development of children’s social competence. These included opportunities for children to reflect on how to provide emotional support to peers in challenging situations (e.g., fear, sadness, anger), engage in collaborative tasks in pairs or small groups, and explore fundamental social values such as kindness, sharing, and respect. Through repeated engagement in these experiences, children were able to practice prosocial behaviors within a supportive and feedback-rich environment, with reinforcement provided by both teachers and peers. Additionally, the program’s narrative structure, built around Timmy’s interactions with a variety of characters, offered emotionally meaningful contexts that illustrated key social–emotional themes, including empathy, cooperation, and inclusion. Narrative-based approaches have been found to be particularly effective in early childhood education for promoting social understanding and enhancing peer-related skills [82,92]. These findings are also consistent with research evaluating the effectiveness of the Preschool PATHS (Promoting Alternative Thinking Strategies) curriculum in early childhood settings [2]. Results indicated that children who received the intervention showed significantly greater improvements in social competence compared to peers in control conditions. Importantly, the success of the intervention was probably moderated by implementation quality and teacher engagement—both of which were key elements in the present study, supported through targeted training and continuous professional support for educators.

No significant intervention effects were observed for internalizing or externalizing difficulties. These non-significant findings may be due to several factors, including the generally low baseline levels of behavioural and emotional problems in the sample, as well as the universal nature of the intervention, which was not specifically tailored to address clinical symptoms. This pattern aligns with prior research showing that universal SEE programs are particularly effective in promoting positive social behaviors, such as prosociality and self-regulation, rather than reducing internalizing or externalizing problems, unless supplemented by targeted or tiered components [13,89,93].

### 6.4. Limitations and Strengths

This study is not without limitations. First, the quasi-experimental design did not allow for full randomization, which may limit the internal validity and the ability to draw definitive causal inferences. However, this methodological choice was necessary to preserve the ecological integrity of the classroom setting and to avoid disrupting the natural organization of school activities. Assigning intact classes to either the experimental or control condition ensured continuity in teacher–child relationships and classroom dynamics, which are essential features of real-world educational interventions [94].

Second, although the study adopted a multi-informant approach by integrating both direct assessments and teacher reports, systematic data from parents were not collected, limiting the ability to capture the full range of children’s social–emotional functioning across home and school contexts. While the intervention included a series of home-based activities designed to foster parent–child interaction and reinforce classroom learning, it was not feasible to systematically monitor the actual implementation of these tasks. Teachers reported that some students returned the completed activities, while others did not, and no standardized procedure was put in place to document the level of family involvement. This decision was made to avoid increasing the workload of educators. As a result, it remains unclear to what extent families engaged with the intervention materials and how this variability may have influenced outcomes. Future studies should incorporate structured parent-report measures and implementation logs to assess fidelity and engagement in home-based components and consider longitudinal follow-ups extending into later school years to capture long-term developmental trajectories.

Third, the intervention was implemented in a limited number of preschools located in specific regions of Northern Italy. This geographic concentration may constrain the generalizability of the findings, as educational practices, classroom dynamics, and socio-cultural norms can differ considerably across regions and countries. Contextual factors such as teacher training availability, institutional support, and local educational policies may have influenced the observed outcomes. Additionally, the relatively small sample size further limits the extent to which the results can be generalized to broader populations. Although the findings provide valuable insights into the effectiveness of the intervention, results should be interpreted with caution when applied to different socio-demographic or educational settings. Future research should aim to replicate these findings in more diverse geographical and socio-economic contexts, with larger samples, to test the robustness, scalability, and cultural adaptability of the intervention.

A final limitation concerns the change in informants between pre- and post-test assessments for teacher-rated measures (SDQ and SCBE-30). Specifically, preschool teachers completed the pre-test evaluations, while primary school teachers completed the post-test assessments. This shift was necessitated by the natural transition of children from preschool to primary school and, thus, reflects a typical developmental trajectory rather than a procedural inconsistency. Nevertheless, this change in informants may introduce variability due to differences in teacher expectations, observational contexts, or relationships with the children. At the same time, it also provides a valuable opportunity to examine children’s socio-emotional development across educational settings and from different adult perspectives, enhancing ecological validity [95].

Nevertheless, the study presents several notable strengths that enhance the validity and relevance of its findings. First, the use of a multi-informant design, combining direct assessments of children’s emotional understanding with teacher-reported evaluations of behaviour, allowed for a more comprehensive and ecologically valid assessment of children’s socio-emotional competencies across different settings [96]. This methodological approach is particularly advantageous in early childhood research, where no single informant can fully capture the complexity of children’s functioning. Direct measures assess children’s skills in a controlled environment, while teacher reports provide insight into how these skills manifest in naturalistic classroom contexts, increasing the external validity of the results.

Second, the longitudinal quasi-experimental design adds significant robustness to the evaluation of program outcomes. Long-term studies in the field of social and emotional education remain relatively scarce, particularly those that extend beyond the immediate post-intervention phase [93]. By assessing children’s outcomes at a later time point, after the transition to primary school, this study provides meaningful evidence regarding the durability of gains. Longitudinal designs are particularly valuable for capturing developmental changes and testing whether early gains are maintained over time, a crucial consideration for educational policy and practice. Additionally, the integration of a home-based component, aligned with classroom activities, likely contributed to the observed outcomes. As supported by prior findings, family engagement enhances the effectiveness of early SEE interventions by reinforcing skill generalization across settings [64,97].

Another key strength of this study lies in its investment in teacher professional development. The training provided to educators was not only foundational for the successful delivery of the program but also represented a sustainable form of capacity-building. Teachers were equipped with theoretical knowledge and practical strategies to promote socio-emotional skills in the classroom, a competence that extends beyond the duration of the intervention. Fostering teachers’ socio-emotional competence is essential for supporting students’ development and well-being, as emotionally competent teachers are better able to build positive relationships, model healthy emotional expression, and manage classroom dynamics effectively [98].

Moreover, research consistently shows that interventions aiming to enhance teachers’ emotional literacy and self-regulation skills contribute to improved classroom climate, greater teacher–student connectedness, and reduced levels of teacher stress and burnout [99,100,101]. These competencies not only increase educators’ self-efficacy in handling emotionally charged situations but also promote their long-term well-being, ultimately benefiting students’ academic and social outcomes [102,103]. Teachers with enhanced social and emotional competencies are more likely to perceive themselves as active agents of change in promoting inclusive, emotionally supportive classroom environments [60,62,104].

Another key factor underlying the program’s sustained impact pertains to the integrated set of developmentally grounded processes embedded within its design. First, the use of narrative-based activities allowed children to emotionally and cognitively engage with the characters, fostering perspective-taking and the development of emotional vocabulary [84,86]. Second, the inclusion of brief, regular mindfulness practices at the beginning of each session helped enhance children’s attentional control and emotional awareness—core components of emotional self-regulation [51,76]. Third, the integration of home-based activities prompted parent–child discussions around emotional experiences, thereby reinforcing the classroom learning within the family context and promoting skill generalization [97]. These multi-component features, embedded in both school and home environments, are consistent with evidence suggesting that interventions that simultaneously target multiple systems (child, educator, and caregiver) are more likely to yield sustained and meaningful outcomes [12,14].

## 7. Conclusions

The present study investigated the long-term effectiveness of *Timmy’s Trip to Planet Earth* program, a school-based intervention grounded in the Social and Emotional Education (SEE) framework [41] and delivered during the final year of preschool, and evaluated 18 months later. The findings provide empirical support for the program’s capacity to enhance foundational social and emotional competencies, with effects observed in both direct and teacher-reported assessments.

Specifically, the intervention group showed significant improvements in the External and Mental components of the Test of Emotion Comprehension (TEC), which assesses children’s ability to recognize facial expressions, to understand how emotions are influenced by external events and mental states, and to regulate emotional expression [65,66]. These results suggest that the program effectively promoted key aspects of emotional understanding and early theory of mind, in line with previous research on emotion knowledge and perspective-taking in early childhood [78].

Children in the experimental group also demonstrated notable improvements in social behaviours, as indicated by a significant increase in teacher-rated prosocial behaviours and social competence, alongside a reduction in conduct problems. These outcomes align with the program’s emphasis on empathy, cooperation, and self-regulation, particularly through narrative and role-play activities that encouraged children to reflect on emotions such as fear and anger and to practice prosocial conflict resolution [86].

Beyond its empirical contributions, the current study holds significant implications for policy, research, and educational practice. First, the findings reinforce the importance of integrating structured, evidence-based SEE curricula into early childhood education as a proactive strategy for fostering long-term socio-emotional development and school readiness. This supports policy directions that advocate for universal mental health promotion starting from the early years, particularly through whole-school and family-inclusive approaches [41,57,58]. Second, the sustained benefits observed 18 months after program implementation emphasize the value of investing in high-quality program design. Key elements include continuity between preschool and early primary education, emotionally rich learning experiences, and sustained professional development for educators [12,17,35,105]. These principles are strongly endorsed by research on effective early transitions and developmental continuity [6]. Third, the study may contribute to the growing body of evidence showing that emotionally supportive learning environments can significantly reduce early disparities in children’s emotional, social, and behavioral functioning—domains that are closely interrelated and predictive of later academic achievement and school adjustment [5,6,18]. By promoting children’s emotional understanding and positive peer interactions, these environments buffer the effects of early disadvantage and prevent the escalation of difficulties that often lead to long-term academic failure and social exclusion.

From a policy perspective, our findings align with European-level recommendations that advocate for the inclusion of SEE as a distinct and mandatory component within national curricula, supported by dedicated resources, teacher training, and continuous monitoring [59]. In particular, policies should prioritize early and universal access to SEE, ensure cultural and contextual adaptation of programs, and reinforce collaboration between schools, families, and external services [64,79]. Moreover, the study supports the implementation of whole-school approaches that embed SEE in both curricular and contextual levels, contributing to school quality, equity, and social cohesion [106]. Finally, for educational research, this study underscores the importance of longitudinal designs that track the developmental impact of early interventions and their systemic conditions of sustainability.

## Figures and Tables

**Table 1:** Overview of learning objectives by core SEE competency areas in *Timmy’s Trip to Planet Earth program*.

SEE Area	SEE Learning Objectives
Self-awareness	To recognize feelings of happiness and sadness in oneself and others To identify how the body reacts to happiness and sadness To connect personal life events to these emotions
Self-management	To identify fear and anger in oneself To connect fear and anger to personal experiences To apply positive strategies to manage fear and anger
Social awareness	To recognize and appreciate individual differences To promote emotional security through acceptance and care To strengthen belonging within the classroom community To support prosocial responsibility and care for others
Social management	To collaborate and share with peers To offer help and support when needed To develop positive and respectful interactions with others

**Table 2:** Demographic characteristics of the study sample at pre-test.

Variable	Experimental (*n* = 45)	Waiting (*n* = 44)	Total (*n* = 89)
Gender			
Female	26	27	43
Male	19	17	46
Mean age (months)	64.27 (3.99)	65.7 (3.48)	64.98 (3.79)
Age range (months)	59–71	59–71	59–71

Note: Standard deviations are presented in parentheses.

**Table 3:** Pre- and post-test means and standard deviations for all instruments and variables by Group Condition.

Instrument	Variable	Pre-Test			Post-Test		
		Experimental (*n* = 45)	Waiting List (*n* = 44)	Total (*N* = 89)	Experimental (*n* = 45)	Waiting List (*n* = 44)	Total (*N* = 89)
TEC	External	2.27 (0.69)	2.23 (0.80)	2.25 (0.74)	2.78 (0.42)	2.23 (0.42)	2.53 (0.58)
	Mental	1.36 (0.61)	1.59 (0.89)	1.47 (0.77)	1.96 (0.82)	1.66 (0.71)	1.81 (0.78)
	Reflective	1.69 (0.85)	1.52 (0.66)	1.61 (0.76)	1.98 (0.72)	1.64 (0.72)	1.81 (0.74)
SDQ	Emotional Problems	1.36 (1.13)	1.84 (2.44)	1.60 (1.89)	0.62 (0.81)	1.73 (1.35)	1.17 (1.24)
	Conduct Problems	0.91 (1.22)	1.14 (1.92)	1.02 (1.6)	0.18 (0.44)	1.2 (1.15)	0.68 (1.01)
	Hyperactivity	1.04 (1.49)	1.45 (2.23)	1.25 (1.9)	1.44 (1.51)	1.79 (1.98)	1.62 (1.76)
	Peer Problems	2.76 (1.48)	3.39 (1.89)	3.07 (1.72)	0.87 (0.94)	1.48 (1.69)	1.16 (1.39)
	Prosocial behaviour	7.89 (1.52)	7.98 (1.61)	7.93 (1.55)	8.29 (1.74)	6.45 (1.58)	7.38 (1.89)
SCBE-30	Social Competence	4.34 (0.76)	4.38 (1.21)	4.36 (1)	4.58 (0.61)	4.13 (0.92)	4.36 (0.8)
	Internalizing Problems	5.37 (0.52)	5.13 (0.91)	5.26 (0.74)	5.36 (0.45)	5.34 (0.65)	5.35 (0.55)
	Externalizing Problems	5.59 (0.42)	5.53 (0.62)	5.56 (0.53)	5.51 (0.46)	5.7 (0.4)	5.6 (0.43)

Note: Standard deviations are presented in parentheses. TEC = Test of Emotion Comprehension; SDQ = Strengths and Difficulties Questionnaire; SCBE-30 = Social Competence and Behavior Evaluation.

## Data Availability

The datasets analyzed during the current study are available from the corresponding author upon reasonable request due to ethical restrictions. Data will be shared with researchers whose proposed use of the data has been approved by all authors, under ethical and privacy considerations.

## Appendix A

**Table A1:** Summary of main effects for outcome variables.

Instrument	Variable	F(1, 87)	*p*-Value	Partial η^2^
TEC	External	7.77	** 0.007 **	0.082
	Mental	8.34	** 0.005 **	0.087
	Reflective	3.94	** 0.05 **	0.043
SDQ	Emotional Problems	3.28	0.074	0.036
	Conduct Problems	4.38	** 0.039 **	0.048
	Hyperactivity	2.87	0.094	0.032
	Peer Problems	91.82	** <0.001 **	0.513
	Prosocial Behaviour	6.23	** 0.014 **	0.067
SCBE-30	Social Competence	0.001	0.972	<0.001
	Internalizing Problems	1.54	0.218	0.017
	Externalizing Problems	0.64	0.427	0.008

Note: Significant *p*-values are shown in bold. TEC = Test of Emotion Comprehension; SDQ = Strengths and Difficulties Questionnaire; SCBE-30 = Social Competence and Behavior Evaluation.

**Table A2:** Summary of group × Time interaction effects for outcome variables.

Instrument	Variable	F(1, 87)	*p*-Value	Partial η^2^
TEC	External	5.44	**0.022**	0.059
	Mental	5.28	** 0.024 **	0.057
	Reflective	0.75	0.39	0.009
SDQ	Emotional Problems	1.76	0.189	0.020
	Conduct Problems	4.38	** 0.014 **	0.068
	Hyperactivity	0.02	0.893	<0.001
	Peer Problems	0.003	0.959	<0.001
	Prosocial Behaviour	18.26	** 0.001 **	0.174
SCBE-30	Social Competence	3.94	** 0.05 **	0.043
	Internalizing Problems	1.95	0.166	0.022
	Externalizing Problems	0.14	0.709	0.002

Note: Significant *p*-values are shown in bold. TEC = Test of Emotion Comprehension; SDQ = Strengths and Difficulties Questionnaire; SCBE-30 = Social Competence and Behavior Evaluation.
